# CRISPR/Cas9-Mediated Disruption of *Xylanase inhibitor protein* (*XIP*) Gene Improved the Dough Quality of Common Wheat

**DOI:** 10.3389/fpls.2022.811668

**Published:** 2022-04-05

**Authors:** Zhengjuan Sun, Mingxia Zhang, Yanrong An, Xu Han, Baojin Guo, Guangde Lv, Yan Zhao, Ying Guo, Sishen Li

**Affiliations:** State Key Laboratory of Crop Biology, College of Agronomy, Shandong Agricultural University, Tai’an, China

**Keywords:** wheat, dough quality, *xylanase inhibitor protein (XIP)*, CRISPR/Cas9, SDS-sedimentation values (SV), stability time (ST)

## Abstract

The wheat dough quality is of great significance for the end-use of flour. Some genes have been cloned for controlling the protein fractions, grain protein content, starch synthase, grain hardness, etc. Using a unigene map of the recombinant inbred lines (RILs) for “TN 18 × LM 6,” we mapped a quantitative trait locus (QTL) for dough stability time (ST) and SDS-sedimentation values (SV) on chromosome 6A (*QSt/Sv-6A-2851*). The peak position of the QTL covered two candidate unigenes, and we speculated that *TraesCS6A02G077000* (a xylanase inhibitor protein) was the primary candidate gene (named the *TaXip* gene). The target loci containing the three homologous genes *TaXip-6A*, *TaXip-6B*, and *TaXip-6D* were edited in the variety “Fielder” by clustered regularly interspaced short palindromic repeats–associated protein 9 (CRISPR/Cas9). Two mutant types in the T_2:3_ generation were obtained (*aaBBDD* and *AAbbdd*) with about 120 plants per type. The SVs of *aaBBDD*, *AAbbdd*, and WT were 31.77, 27.30, and 20.08 ml, respectively. The SVs of the *aaBBDD* and *AAbbdd* were all significantly higher than those of the wild type (WT), and the *aaBBDD* was significantly higher than the *AAbbdd*. The STs of *aaBBDD*, *AAbbdd*, and WT were 2.60, 2.24, and 2.25 min, respectively. The ST for the *aaBBDD* was significantly higher than that for WT and was not significantly different between WT and *AAbbdd*. The above results indicated that XIP *in vivo* can significantly affect wheat dough quality. The selection of *TaXip* gene should be a new strategy for developing high-quality varieties in wheat breeding programs.

## Introduction

Wheat (*Triticum aestivum*. L) is the main source of nutrition and feeds more than 30% of the world’s population (Rasheed et al., 2018; Wang et al., 2019). The demand for wheat with high-quality attributes has increased globally due to the growing population and the rising living standards in countries worldwide (Peña et al., 2008; Kumar et al., 2019). The presence of wheat gluten gives the dough viscoelasticity and ductility, and it can be processed into a variety of foods to meet people’s needs (Veraverbeke and Delcour, 2002).

The stability time (ST) and SDS-sedimentation value (SV) are the key quality traits for wheat and play a critical role in determining end-use products. ST is the main parameter of the farinograph, which determines the final quality of bread, steamed bread, noodles, and other wheat foods (Tsilo et al., 2011). The length of the ST reflects the resistance of the dough to kneading, and doughs with high ST values have good flexibility and high gluten strength (Veraverbeke and Delcour, 2002; Dangi et al., 2019). The SV can be used as an essential indicator for detecting the quality of gluten, which positively correlates with dough rheological properties (Kaur et al., 2013). In general, the strong flour demands that the ST and SV are greater than 8 min and 40 ml, respectively (Ma et al., 2021). Moreover, because the mensuration of SV is simple and convenient and uses less flour, it is usually used as an indicator of early generation selection in wheat quality breeding programs (Peña et al., 2008).

The ST and SV are parameters highly influenced by the protein compositions, i.e., glutenins and gliadins, their fractions, the ratio between them as well as the total protein content (Payne et al., 1988; Deng et al., 2006; Rogers et al., 2006; Caballero et al., 2008; Dhaka and Khatkar, 2015). Some genes have been cloned in controlling the protein compositions, high molecular weight glutenin subunits (HMW-GS; Thompson et al., 1985; Anderson and Greene, 1989), low molecular weight glutenin subunits (LMW-GS) (D’Ovidio et al., 1992; Ikeda et al., 2002), and gliadin (Rafalski et al., 1984; Sugiyama et al., 1986). The genes regulated other wheat quality traits, such as grain protein content (Uauy et al., 2006; Hagenblad et al., 2012) and grain hardness (Gautier et al., 1994; Chen et al., 2006), were also cloned. Some transcription factors, such as *TaNAC019*, directly activated the expression of HMW-GS genes by binding to a specific motif in their promoters and interacting with the *TaGlu-1* regulator *TaGAM* (Gao et al., 2021).

Cereals contain proteinaceous inhibitors of endo-xylanases (McLauchlan et al., 1999). The inhibitors of xylanase in wheat are grouped into three classes: TAXI (*Triticum aestivum* xylanase inhibitor; Debyser et al., 1997, 1999), XIP (xylanase inhibitory protein; McLauchlan et al., 1999; Elliott et al., 2003), and TLXI (thaumatin-like xylanase inhibitor; McLauchlan et al., 1999). The XIP-type xylanase inhibitor genes are responsible for plant defense (Takahashi-Ando et al., 2007). The effect of the XIP *in vivo* on the grain quality of wheat has not been reported.

Gene editing is an important tool to study gene function. In the past few years, clustered regularly interspersed short palindromic repeats (CRISPR) have achieved the ability to control the specific introduction of directed sequence variation (Gilbert et al., 2013; Shan et al., 2013; Soda et al., 2018). This technology has been applied to the genetic studies of wheat quality. Zhang S. J. et al. (2018) used CRISPR/Cas9 to silence HWM-GS in wheat, which significantly reduced dough strength and bread-baking quality. Sánchez-León et al. (2018) found that CRISPR/Cas9 could be used to produce low-gluten foodstuff and serve as source material to introgress this trait into elite wheat varieties. Li et al. (2020) edited *TaSBEIIa* in both winter and spring wheat varieties by CRISPR/Cas9 modification of starch composition, structure, and properties.

In this study, we found a quantitative trait locus (QTL) under multi-environments for the ST and SV, *QSt/Sv-6A-2851*, on chromosome 6A using TL-recombinant inbred lines (RIL) population. This locus contains the *xylanase inhibitor protein* (*Xip*) gene. We performed functional validation of the *TaXip* gene using the CRISPR/Cas9 mutagenesis system.

## Materials and Methods

### RIL Population, Field Trials, and Quality Trait Measurements

The RIL population of 184 lines used in the study was derived by single-seed descent (SSD) methods from a cross of “TN18 × LM6” (TL-RIL, F_11_ in 2015; Zhang et al., 2019). TN18 is a cultivated variety developed by our research group and LM6 is an excellent line developed by the Linyi Academy of Agricultural Sciences. The field trials were conducted by Guo et al. (2020) at the experimental farm of Shandong Agricultural University in Tai’an for three growing seasons with two replications: 2011–2012 (E1), 2012–2013 (E2), and 2013–2014 (E3).

The seed samples for the TL-RIL population obtained from the harvested grains were stored at room temperature for approximately three months and then milled using a Bühler experimental mill (Bühler mill, Bühler-Miag Company, Braunschweig, Germany; Guo et al., 2020). The flour was used to test the SV and farinograph parameters including ST. The SV was determined with a sedimentation volume instrument (BAU-A type) and the farinograph parameters were determined by a farinograph (Brabender GmbH and CoKG).

### The Genetic Map of Unigenes and Quantitative Trait Locus Analysis for the TL-RILs

By RNA sequencing each line of the TL-RIL population, a genetic map of unigenes based on the physical position in reference genome RefSeq v1.1 (IWGSC, 2018) was previously constructed by our group (Zhang, 2019). The map included 27,452 sites; 28,811 unigenes; 31,445 sub-unigenes; and 117,758 SNP/InDels. Using the unigene map and phenotypic data (Guo et al., 2020), we mapped QTLs by IciMapping4.1 and MapQTL5.0 software. For IciMapping4.1, inclusive composite interval mapping (ICIM) was carried out with a step size of 0.5 cm. The parameter for handling missing phenotypic data was “Deletion.” For MapQTL 5.0, the multiple-QTL model (MQM) package with a mapping step size of 0.5 cm was used to map QTLs. The LOD threshold for declaring a significant QTL in both the software was a LOD > 2.5.

### DNA and RNA Extraction and Primer Design

For the variety “Fielder” and the progenies of gene editing, total DNA was extracted using a DNA extraction kit (Tiangen, Beijing, China). The quality and concentration of the total DNA were determined using a NanoDrop 2000c spectrophotometer (Thermo Fisher Scientific, Wilmington, DE, United States). Total RNA was extracted using an AG21019 RNA extraction kit (Accurate, Changsha, China), digested with DNase to remove residual DNA, and reverse transcribed into cDNA using a Prime Script TMRT-PCR kit AG11711 (Accurate, Changsha, China). The quality and concentration of the total RNA were determined using 1% agarose gel electrophoresis and a NanoDrop 2000c spectrophotometer (Thermo, Wilmington, DE, United States). Genomic DNA was extracted using the Tiangen DNA quick Plant System (Tiangen, Beijing, China) for deep sequencing.

### Amplification of the *TaXip* Genes

Total DNA and cDNA were used to amplify *TaXip*-6A, *TaXip*-6B, and *TaXip*-6D. Sequence amplification was performed with FastPfu high-fidelity enzyme (TransGen Biotech, Beijing, China). The primers were TAXIP6A-F: ccttaggattcactcctgcg; TAXIP6A-R: gttccgagtggtgatcagc; TAXIP6B-F: gcgctagagcagag gatcctaac; TAXIP6B-R: ggcttgtggaagcatagctcc; TAXIP6B-R: gtc ggatacgaattggcg; and TAXIP6D-R: aactgtgcgaccaatctgttc). PCR was run at 95°C for 5 min, followed by 33 cycles of denaturing at 94°C, annealing for 30 s, and extension at 72 C for 60 s, with a final extension at 72°C for 5 min on an ABI PCR system 2400. The PCR products were separated by electrophoresis, recovered, and ligated to a *pEASY*^®^-Blunt Cloning Kit (TransGen Biotech, Beijing, China). They were transformed into *E. coli* competent cells and selected on LB plates containing 50 μg/ml ampicillin. Positive clones were sequenced. Complete multiple alignments of sequences (Supplementary Figure 1) and translations of nucleotides into amino acid sequences were performed by DNAMAN Version 5.0 (Gong et al., 2013).

### Acquisition and Culture of Gene Editing Plants

The sequences of the *TaXip* genes were obtained from the cloned sequence from “Fielder” and used to design sgRNA target sequences in CRISPR-direct^1^ and CRISPOR.^2^ The sgRNA2 was 5′-GTCCAACCGCTCCGCGCTCG-3′ from 374 to 393 bp (start at ATG) with protospacer adjacent motif (PAM) site CCC, and sgRNA1 was 5′-ACAACATCCGCGGCGGCCCG-3′ from 550 to 569 bp with PAM site GGG.

Using the designed primer (MT1T2-XF_0_: gacaacatccgcggcg gcccggttttagagctagaaatagc; MT1T2-XR_0_: gtccaaccgctccgcgctcgcgc ttcttggtgcc; MT1T2-XF: aataatggtctcaggcgacaacatccgcggcggcccg; and MT1T2-XR: attattggtctctaaacgtccaaccgctccgcgctcg) and *MT1T2* vector as templates, PCR amplification was conducted to obtain the intermediate vector fragment containing two sgRNAs (964 bp). The sgRNAs were connected to the *pBUE411* vector by an enzymatic ligation reaction. The schematic map of the binary vector is shown in Supplementary Figure 2. The RB/LB represent the left and right borders of the vector; *TaU3P* was the wheat U3 gene promoter and used to drive sgRNAs; sgRNA site refers to the guide RNA clone site; sgRNA SC was the sgRNA scaffold; *PUbi* was the ubiquitin gene promoter and used to drive *zCas9*; *zCas9* was the maize codon-optimized Cas9; *Tnos* was the Nos terminator; and *P35S* was the 35S promoter to drive bar (Xing et al., 2014). After sequencing the target sites, the binary vector was transformed into the wheat variety “Fielder” by *Agrobacterium tumefaciens*-mediated transformation (Zhang S. J. et al., 2018). A total of 29 T_0_ plants were yielded in August 2019. In the greenhouse of Shandong Agricultural University, T_0_ generation seeds of different genotypes were selected and T_1_ plants were planted. After identifying the mutant types of T_2_ plants, the seeds of T_2_ mutant plants were sown in flowerpots with 4 plants in each flowerpot and 30 flowerpots for each edited type (T_2:3_ lines). The seeds harvested in the T_2_ generation were the T_2:3_ lines that were used for ST and SV measurements. All plants were grown in the greenhouse with 16 h of light and 8 h of darkness at 25°C. The grains from WT plants were similarly harvested and served as controls in the experiments.

Genomic DNA was extracted from the leaves of the genome-edited plants. To identify mutation types of T_0_, T_1_, and T_2_ generations, specific primers (g6aF: ggagtgagtac ggtgtgcGTTGGCGGCTACGGCACC; g6aR: gagttggatgctggatg gCACCGGACCGTCGCCGT; g6bF: ggagtgagtacggtgtgcATCG GCGGCTACGGCACC; g6bR: gagttggatgctggatggCACCGGAC CGTCGCCGTT; g6dF: ggagtgagtacggtgtgcCATCGGCGGCTAC GGCG; g6dR: gagttggatgctggatggCGGACCGTCGCCGTCAG GT) were designed to sequence the target regions of the A, B, and D genomes. Then, the PCR products were amplified and sequenced by Hi-Tom (Liu et al., 2019). Next-generation sequencing technology was used to detect the mutation sites. Li et al. (2021) reported that the lowest average ratio for plants with albino phenotype was over 80% after the *TaPDS* gene was edited. This indicated an editing threshold for displaying loss of function phenotype. We used this strategy to classify different genotypes.

### Sedimentation Values and Stability Time Determination and Statistical Analysis for Mutant Lines and “Fielder”

For the variety “Fielder” (wild type, WT) and the T_2:3_ mutant lines, about 500 g grains were obtained from mixed samples of 120 individual plants, respectively. The flour was milled by a small experimental mill of Quadrumat Junior (Brabender GmbH and CoKG, Germany) with no xylanases added and then passed through a 80-mesh sieve. The separate flour samples were used to test the SV and ST. The SV was tested based on Peña et al. (1990) and corrected to 14% flour moisture content. The ST is a farinograph parameter which was determined using Automatic Farinograph-AT (Brabender GmbH and CoKG, Germany) referred to Tian (2006).

SPSS 17.0 software (SPSS, Chicago, IL, United States) was used for statistical analysis of the ST and SV. Multiple comparisons using LSD to identify where the differences lay. *P*-value less than 0.05 or 0.01was considered as significant or extremely significant.

## Results

### Acquisition of the Candidate Gene of the Stability Time and Sedimentation Values

**FIGURE 1:**
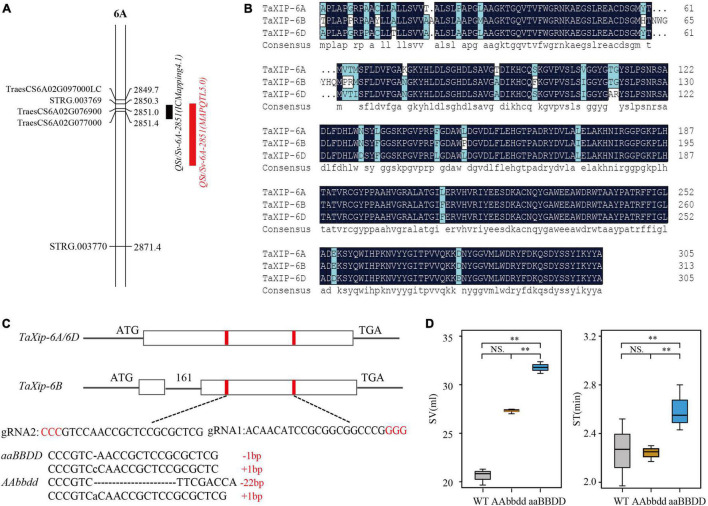
Mapping of *QSt/Sv-6A-2851* and development of *TaXIP* mutants by CRISPR/Cas9-mediated genome editing. **(A)** QTL mapping for *QSt/Sv-6A-2851* using IciMapping 4.1 (black bar), MapQTL5.0 (red bar) software. **(B)** Amino acid sequence alignment for three homologous genes *TaXip-6A*, *TaXip-6B*, and *TaXip-6D* in “Fielder.” **(C)** Schematic map of the sgRNAs target site selection in the *TaXip* genes and the T_2:3_ mutant types (*aaBBDD* and *AAbbdd*) induced by CRISPR/Cas9. The vertical bar is exon and the horizontal line is intron, the protospacer-adjacent motif (PAM) is highlighted in red. **(D)** Difference for SV and ST between T_2:3_ mutant lines and WT (Fielder). “**”represents extremely significant difference.

Using the TL-RIL population and their genetic map of unigenes based on RNA-Seq technology (Zhang, 2019), we mapped the QTLs for quality traits. Of these, a QTL for ST and SV, *QSt/Sv-6A-2851*, was detected under multi-environments with peak positions ranging from 2850.3 to 2860.4 cM by IciMapping 4.1 and MapQTL 5.0 software (Figure 1A and Supplementary Table 1). The peak position of *QSt/Sv-6A-2851* covered *STRG.003769* and two annotated unigenes in RefSeq v1.1 (IWGSC, 2018), *TraesCS6A02G076900* (46974751–46979670 bp) and *TraesCS6A02G077000* (47104969–47106326 bp). The *STRG.003769* is a transcript with no complete gene structure. The *TraesCS6A02G076900* was annotated as an ABC-2-type transporter family protein. The *TraesCS6A02G077000* was annotated as a xylanase inhibitor protein (named the *TaXip* gene). Sequence analysis revealed that *TraesCS6A02G076900* had three SNPs in the 5′UTR, two SNPs in the intron region, and one SNP in the 3′UTR without amino acid changes. But the *TraesCS6A02G077000* had one SNP at the 390 bp (start at ATG) in the exon region that led to amino acid changes (cysteine to tryptophan). Additionally, our group found a QTL cluster that contained SV and ST on chromosome 6A between *TraesCS6A02G070900* and *TraesCS6A02G125200* using the same TL-RIL population and its molecular marker map (Guo et al., 2020). Xylanases are widely used as additives in the baking industry to improve processing and product quality (Goesaert et al., 2005). The xylanase inhibitor protein is likely to inhibit the decomposition of xylanase *in vivo* (Simpson et al., 2003). Therefore, we speculated that *TraesCS6A02G077000* was the candidate gene of the QTL *QSt/Sv-6A-2851*.

### Amplification of the *TaXip* Homologous Genes

Three homologs of *TaXip* were identified by BLAST analysis in the IWGSC database^3^ : *TaXip-6A* (*TraesCS6A02G077000*), *TaXip-6B* (*TraesCS6B02G103900*), and *TaXip-6D* (*TraesCSU02G026500*). These homologous genes were amplified separately in the parents TN18 and LM6 of the TL-RILs and the variety “Fielder” (used in gene editing). For TN18 and LM6, an SNP site at the 390 bp was found in the *TaXip-6A* gene. It was base C in TN18 and G in LM6, encoding cysteine and tryptophan, respectively, which is in accordance with the RNA-Seq result. The exon sequences of *TaXip-6B* and *TaXip-6D* were not different between the parents. In “Fielder,” *TaXip-6A* and *TaXip-6D* have only one exon with 915 bp open reading frames and encode 305 amino acids. In contrast, *TaXip-6B* has one intron and two exons, 939 bp open reading frames, and encodes 313 amino acids (Figure 1B). Based on the amino acid sequences and domains, paired comparison results showed that the identities between *TaXip-6A* and *TaXip-6D*, between *TaXip-6A* and *TaXip-6B*, and between *TaXip-6B* and *TaXip-6D* were 94.75, 91.69, and 91.37%, respectively.

### Obtaining of Gene Editing Plants by CRISPR/Cas9

The sgRNA targets for *TaXip-6A*, *TaXip-6B*, and *TaXip-6D* were designed based on the conserved domains in all the three sub-genomes. Two sgRNAs (sgRNA1 and sgRNA2) were designed to target the first exon on *TaXip-6A* and *TaXip-6D*, the second exon on *TaXip-6B* (Figure 1C). The *pBUE411* vector was designed to create In/Del in the fourth base after PAM. In the T_0_ generation, a total of 29 mutated plants were identified with 7, 7, 5, and 10 mutant plants that were edited for A, D, AB, and ABD sub-genome(s), respectively. In the T_2:3_ generation, two genotypes of mutants were obtained (Figure 1C): the *aaBBDD* genotype with an editing ratio greater than 80% for subgenome A and less than 20% for B and D, and the *AAbbdd* genotype with an editing ratio greater than 80% for subgenomes B and D and less than 20% for A. In the *aaBBDD*, 1 bp was deleted or 1 bp was inserted for 6A and in the *AAbbdd* 22 bp was deleted for 6B and 1 bp was inserted for 6D (Figure 1C). After protein prediction,^4^ we found that all these mutations would lead to frameshift mutations and make the termination codon appear in advance, leading to protein functional inactivation (Supplementary Figure 3).

### Changes for the SV and ST Between Wild and Mutant Genotypes

The SVs of the two mutant genotypes, *aaBBDD* and *AAbbdd*, and WT were 31.77, 27.30, and 20.08 ml, respectively (Supplementary Table 2). The SVs of *aaBBDD* and *AAbbdd* were significantly higher than that of the WT (Figure 1D); the SV of *aaBBDD* was significantly larger than that of *AAbbdd* and WT; and the SV of *AAbbdd* was significantly larger than that of WT. These results indicated that *TaXip-6A, TaXip-6B*, and *TaXip-6D* significantly influenced the SV, but the effect of *TaXip-6A* was greater than those of *TaXip-6B* and *TaXip-6D.* The STs of *aaBBDD*, *AAbbdd*, and WT were 2.60, 2.24, and 2.25 min, respectively (Supplementary Table 3). The ST of the mutant genotype *aaBBDD* was significantly higher than that of WT and *AAbbdd*, but was not significantly different between WT and *AAbbdd* (Figure 1D). This indicates that *TaXip-6A* significantly affected the ST, while *TaXip-6B* and *TaXip-6D* have little effect on the ST.

## Discussion

**FIGURE 2:**
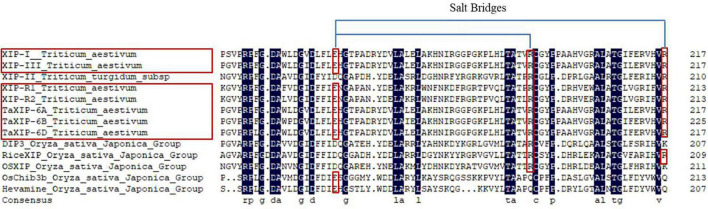
Comparison of the amino acid residues surrounding the chitinase “active site” region of XIPs and chitinase. Conserved amino acid residues are shaded. The two Arg residues (red boxed) and the catalytic Glu residue are joined by blue lines. The names of the sequences with salt bridges are boxed in red. Genbank accession numbers are as follows: XIP-I: CAD19479.1; XIP-II: CAC87260.1; XIP-III: BAD99103.1; XIP-R1: BAF74363.1; XIP-R2: BAF74364.1; RiceXIP: BAG89082.1; DIP3: AFM95334.1; OSXIP: AXF92897.1; OsChib3b: BAA22266.1.

For the genes of XIP class, *Xip-I*, *Xip-III*, *Xip-R1*, *Xip-R2*, *Xip-II*, and *xip-9023*, *xip-366* (Elliott et al., 2002, 2009; Igawa et al., 2005; Takahashi-Ando et al., 2007; Liu et al., 2017) have been cloned in wheat with the functions of plant defense by binding glycoside hydrolase families 10 (GH10) and 11 (GH11; Payan et al., 2004). TaXIP-6A was most similar to wheat XIP-III, XIP-I, and rice acidic class III chitinase (Park et al., 2002) with a protein sequence identity of 98.69, 87.25, and 36.59%, respectively. The sequence alignment of TaXIP-6A, TaXIP-6B, and TaXIP-6D conservation of the two Arg residues (Figure 2; red boxed) proved to be engaged in salt bridges with the catalytic Glu residue (Figure 2; blue bold and red boxed) in XIP-I. This feature suggests that TaXIP-6A, TaXIP-6B, and TaXIP-6D lack chitinolytic activity, as demonstrated in the case of XIP-I (Payan et al., 2003). It indicated that TaXIP functions as an inhibitor of xylanase. The *TaXip-6A* gene was expressed notably in the grain at the later stage of grain development.^5^ In this study, we indicated that the knock-out XIP *in vivo* significantly affected the SV and ST. That is to say, the endogenous XIP affected dough quality. The addition of fungal xylanase *in vitro* can improve the processing quality of dough and end-product quality of bread (Wang et al., 2005; Ghoshal et al., 2013, 2017; Ahmad et al., 2014; Leys et al., 2019). So we speculated that the SV and ST, as important indicators for dough quality, may be affected by XIP through xylanase. But what is the mechanism of XIP and xylanase interaction, and how the xylanase improves the dough quality needs to be further studied.

The factors affecting wheat quality have mainly been focused on protein (Sharma et al., 2020), starch (Rakszegi et al., 2006), and fat (Bonnand-Ducasse et al., 2010). HMW-GS is one of the most important factors affecting dough rheological properties and bread-making quality. Payne et al. (1980, 1987) established a standard of *Glu-1* quality score based on SV that was widely used to evaluate the quality of wheat varieties (Weegels et al., 1996; Gianibelli et al., 2001; Esmaeilzadeh Moghaddam et al., 2011). Ram et al. (2015) found that *Glu-B1i* and *Glu-D1d* showed a highly significant positive effect (*P* < 0.001) on SV and also had additive effects. The allelic genes of *Glu-1* caused the SV variations of several or more than 10 units (ml) (Wang et al., 2018; Chen et al., 2019). The SV decreased by about 22 ml by silencing the five homologous *Glu-1* genes (1Ax2*, *1Bx7, 1By9, 1Dx5*, and *1Dy10*) (Zhang Y. J. et al., 2018). In this study, the SVs of *aaBBDD* and *AAbbdd* were 11.69 and 7.22 ml higher than WT, respectively. The allelic gene selection of XIP should be a new strategy for developing high-quality varieties of wheat.

## Data Availability Statement

The original contributions presented in the study are included in the article/Supplementary Material, further inquiries can be directed to the corresponding authors.

## Author Contributions

SL contributed to the conception of the study. ZS performed the experiment and the data analyses. MZ and BG performed the unigene map construction. ZS and YG contributed to the quality mensuration. ZS, SL, and YA wrote the manuscript. XH, GL, and YZ assisted in gene editing and plant culture. All authors read and approved the manuscript.

## Conflict of Interest

The authors declare that the research was conducted in the absence of any commercial or financial relationships that could be construed as a potential conflict of interest.

## Publisher’s Note

All claims expressed in this article are solely those of the authors and do not necessarily represent those of their affiliated organizations, or those of the publisher, the editors and the reviewers. Any product that may be evaluated in this article, or claim that may be made by its manufacturer, is not guaranteed or endorsed by the publisher.

## Footnotes

1
http://crispr.dbcls.jp/

2
http://crispor.tefor.net/

3
http://plants.ensembl.org/Triticum_aestivum/Info/Index

4
http://www.detaibio.com/sms2/translate.html

5
http://www.wheat-expression.com/
