# Supplementary material for: CRISPR/Cas9-Mediated Disruption of Xylanase inhibitor protein (XIP) Gene Improved the Dough Quality of Common Wheat
Source: Front Plant Sci. 2022 Apr 5;13:811668. doi: 10.3389/fpls.2022.811668 (PMC9018002; doi:10.3389/fpls.2022.811668)
Supplement: Supplementary file 1 [file Data_Sheet_1.docx]

Supplementary Material

**Supplementary Figures**

**
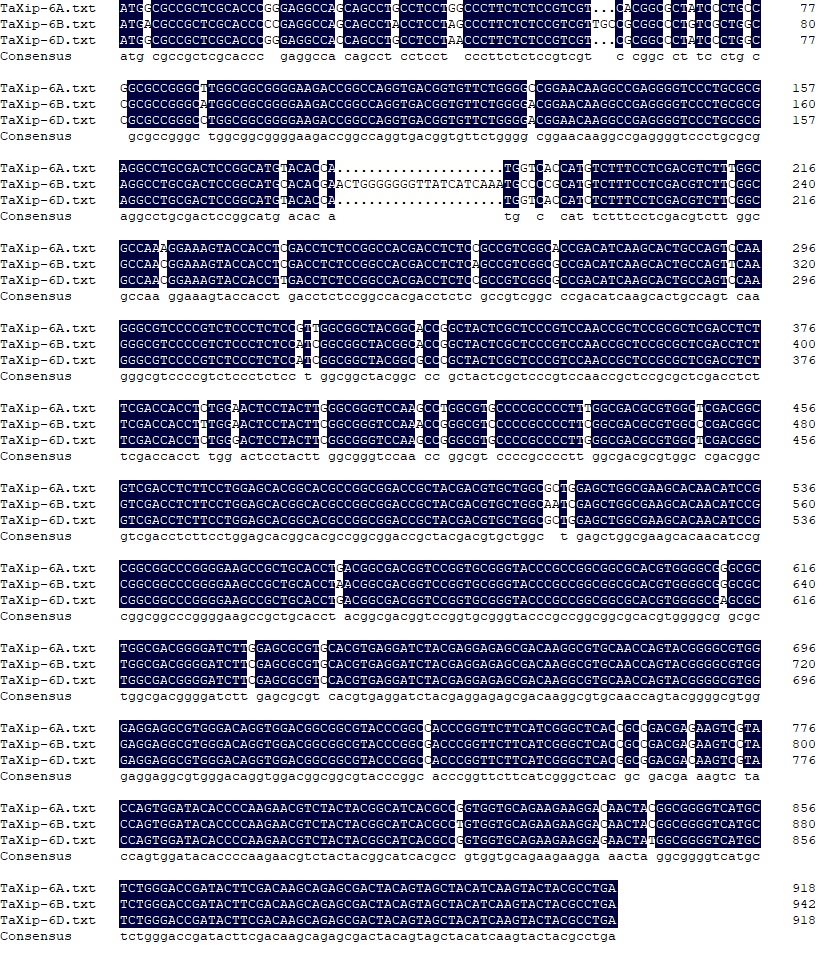
**

**Supplementary Figure 1 Coding sequences alignment of *TaXip-6A, TaXip-6B* and *TaXip-6D* in Fielder**


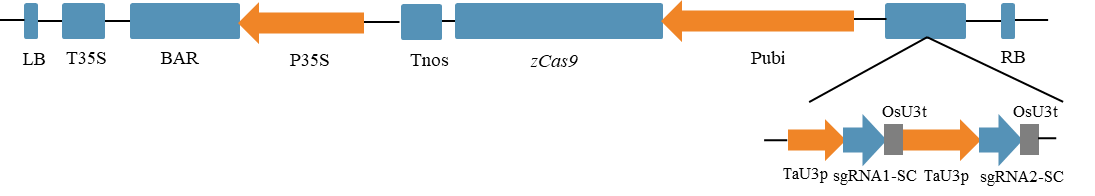


**Supplementary Figure 2 Schematic map of the binary vector of the *TaXip* gene for genome editing**

RB/LB represent the left and right borders of the vector; *TaU3P* was the wheat U3 gene promoter; sgRNA site refers to the guide RNA clone site; sgRNA SC was the sgRNA scaffold; *PUbi* was the ubiquitin gene promoter; *zCas9* was the maize codon optimized Cas9; *Tnos* was the Nos terminator; *P35S* was the 35S promoter.

**
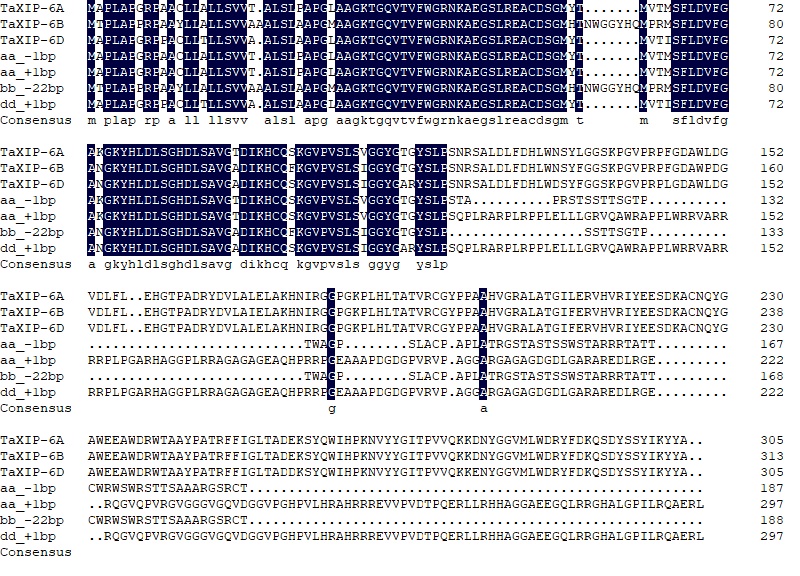
**

**Supplementary Figure 3 Amino acid changes for T_2:3_ mutant lines**

**Supplementary Tables**

**Supplementary Table 1 QTL location for *QSt/Sv-6A-2851* using Icimapping 4.1 and MapQTL5.0 Software**

| **Software** | **Trait** | **Environment** | **Position (cM)** | **LOD** | **Rsq (%)** | **Additive effect** |
| --- | --- | --- | --- | --- | --- | --- |
| **ICMapping4.1** | ST | E13 | 2851.0 | 4.36 | 8.2 | -1.19 |
|  | SV | E14 | 2850.5 | 28.58 | 18.3 | -3.25 |
| **MAPQTL5.0** | ST | AV | 2858.4 | 3.01 | 8.8 | -1.00 |
|  | ST | E13 | 2859.4 | 4.64 | 11.8 | -1.73 |
|  | ST | E12 | 2860.4 | 2.73 | 7.3 | -1.07 |
|  | SV | E14 | 2850.3 | 3.81 | 9.2 | -1.14 |
|  | SV | E12 | 2858.4 | 5.73 | 14.5 | -1.26 |

**Supplementary Table 2 SV and ST values in WT (Fielder) and T_2:3_ gene mutant lines**

| Genotype | Stability time (min) | SDS-sedimentation value (mL) |
| --- | --- | --- |
| WT | 2.25 | 20.08 |
| aaBBDD | 2.60 | 31.77 |
| AAbbdd | 2.24 | 27.30 |
